# The human monkeypox in Saudi Arabia and global tourism

**DOI:** 10.1016/j.amsu.2022.104686

**Published:** 2022-09-17

**Authors:** Najim Z. Alshahrani, Abdullah M. Assiri, Jaffar A. Al-Tawfiq, Alfonso J. Rodriguez-Morales, Ranjit Sah

**Affiliations:** Department of Family and Community Medicine, Faculty of Medicine, University of Jeddah, Jeddah, Saudi Arabia; Ministry of Health, P.O Box 11461, Riyadh, 11176, Saudi Arabia; Infectious Disease Division, Department of Medicine, Indiana University School of Medicine, Indianapolis, IN46202, USA; Infectious Disease Division, Department of Medicine, Johns Hopkins University School of Medicine, Baltimore, MD21218, USA; Prince Abdullah Bin Khaled Coeliac Disease Research Chair, King Saud University, Riyadh, 11362, Saudi Arabia; Faculty of Medicine, Institución Universitaria Visión de las Américas, Pereira, Risaralda, Colombia; Grupo de Investigación Biomedicina, Faculty of Medicine, Fundación Universitaria Autónoma de las Américas, Pereira, Risaralda, Colombia; Clinical Epidemiology and Biostatistics, Universidad Cientifica Del Sur, Lima, Peru; Tribhuvan University Teaching Hospital, Institute of Medicine, Kathmandu, Nepal

The world is still recovering from the COVID-19 pandemic, and thus another epidemic of viral infection would cause undue stress on the healthcare system. On the other hand, the COVID-19 pandemic has made the world better prepared for future epidemics. Thus, no surprise that the response to monkeypox outbreaks in various regions around the global has been brisk. Nonetheless, there are reasons to worry, as the virus has spread quickly, resulting in almost 20,000 confirmed cases globally in less than three months [1]. Moreover, cases are being reported from countries that have historically not reported monkeypox, including Saudi Arabia [1]. By August 2022, Saudi Arabia had already registered five confirmed cases [2]. Although the numbers are not high, worrisome are the figures emerging from EU nations like Germany, Spain, Portugal, and France [3].

Early outbreaks of this virus were identified in 1970 in the Democratic Republic of Congo, where it remains endemic. However, its infrequent outbreaks have been reported outside Africa in various countries like the United States (US) 2003 outbreak and in the United Kingdom (UK), Singapore, and more [4,5]. In all previous outbreaks, the infection was imported and related to travel to the endemic zones, and the related health authorities successfully contained the virus. However, the present outbreak quickly spread to various parts of the world and thus became a cause of concern and leading the World Health Organization (WHO) to declare this disease as a Public Health Emergency of International Concern (PHEIC) on July 23, 2022.

Monkeypox is a zoonotic disease. It is a double-stranded DNA virus belonging to the Poxviridae family. There are two distinct known types of the virus: the west African clade and the central African (Congo Basin). Although the virus mainly spreads from animal to human, its human-to-human spread is also possible. It is more likely to spread by coming in close contact with someone infected, like when having sex. However, it may also spread via respiratory secretions [4]. The present global spread indicates that human-to-human is the primary mode of disease transmission. It has an incubation period of about a week, though it could be up to three weeks. It causes self-limiting illness in most, causing fever, intense headaches, lymphadenopathy, and myalgia, along with skin eruptions appearing 1–3 days after the appearance of fever. In most, it would cause a self-limiting illness lasting 2–4 weeks. However, previous reports indicate that it has a fatality rate of 3–6% [4]. So far, from the multi-country outbreak outside Africa in 2022, three deaths have been confirmed up to July 30, 2022; two in Spain and one in Brazil (occurring among immunosuppressed patients). The present outbreak is due to the West African clade (Clade 3, mainly linage B.1), which causes less severe disease [3].

Regarding Saudi Arabia, all reported cases were among those with a history of visiting EU nations [2]. However, Saudi Arabia remains at high risk, not only due to continued travel to the EU nations but also for other reasons like massive inflow of tourists, trade in exotic animals, and even proximity to the African continent. Additionally, studies suggest that the nature of monkeypox epidemiology is changing, with a greater number of cases reported in middle-aged and older adults, unlike earlier outbreaks. It is explained by the waning immunity to the pox virus, as immunization to smallpox seized in the early 1980s. This may also explain the scale of the current outbreak and why it poses a greater threat [6].

There are other concerns, primarily the lack of sufficient knowledge about the pathogen among general population and healthcare workers [7]. Studies of earlier outbreaks, like the US, show doctors are not well-prepared to identify monkeypox infection. The US outbreak of 2003 was partially the result of the late identification of the causative agent [5]. Findings in Saudi Arabia are not encouraging. A study done in Saudi Arabia through an online survey among young practitioners found that only 18.6% of the surveyed medical practitioners reported having some understanding of managing monkeypox [8]. Studies also suggest a significant global knowledge gap, as more cases are reported in individuals with no travel history [9]. There are also worries that as the infection spreads, the pathogen may mutate, resulting in a shift in virulence. Since this shift in virulence has much to do with transmission and time scales, controlling this outbreak in its early stages is essential [10]. However, controlling this outbreak is only possible through a joint global initiative.

Regarding countering the threat, the WHO has declared it a health emergency, and Saudi MOH has also come up with guidelines for preventing the spread of the infection. Among prevention measures is timely screening, with nucleic acid amplification testing (NAAT) using conventional polymerase chain reaction (PCR) methods. In addition, isolating and tracing the contacts of individuals affected by the infection and targeted use of the second- or third-generation smallpox or monkeypox vaccines [11].

Besides the disease prevention guidelines, Saudi MOH has also developed guidelines for supportive care and treatment of confirmed monkeypox cases. At present, along with supportive care like managing rashes, preventing their secondary infection, and countering oral sores, conjunctivitis, and dehydration, the MOH also has a recommendation for the pharmacotherapy of the infection. At present, Saudi MOH recommends using Brincidofovir as an antiviral agent or Vaccinia immune globulin (SPIG) [11]. In addition, CDC US have also identified other antiviral agents like Tecovirimat and Cidofovir [12].

Currently, the public health threat posed by monkeypox remains moderately high in Saudi Arabia and globally. There are possibilities that this epidemic would have only limited reach, like earlier outbreaks. Nonetheless, considering its global spread, it is essential to be prepared. Many tools, like vaccines and antiviral drugs, are already available to prevent and manage the infection. However, the availability of these tools is a significant issue in Saudi and globally. Additionally, there is an issue of knowledge gap among healthcare workers, necessitating more severe public health measures and the development of disease prevention and management guidelines.

## Ethical approval

Not applicable.

## Sources of funding

No funding received.

## Author contributions

NZA design and draw the original draft, AMA, JMAT, AJRM and RS review the literature, critically edit the manuscript. All authors read and approve the final manuscript.

## Declaration of competing interest

No conflicts of interest.

## Research registration Unique Identifying number (UIN)

1. Name of the registry: N/A
2. Unique Identifying number or registration ID: N/A
3. Hyperlink to your specific registration (must be publicly accessible and will be checked): N/A

## Guarantor

Najim Z. Alshahrani.

Abdullah M. Assiri, Jaffar A. Al-Tawfiq, Alfonso J. Rodriguez-Morales, Ranjit Sah.

## References

[1] 2022 monkeypox outbreak global map | monkeypox | poxvirus | CDC. https://www.cdc.gov/poxvirus/monkeypox/response/2022/world-map.html.

[2] (Jul. 25, 2022). http://saudigazette.com.sa/article/623301.

[3] (Jun. 15, 2022). Epidemiological Update: Monkeypox Multi-Country Outbreak.

[4] Monkeypox. https://www.who.int/news-room/fact-sheets/detail/monkeypox.

[5] Ligon B.L. (2004). Monkeypox: a review of the history and emergence in the Western hemisphere. Semin. Pediatr. Infect. Dis..

[6] Bunge E.M. (2022). The changing epidemiology of human monkeypox—a potential threat? A systematic review. PLoS Neglected Trop. Dis..

[7] Alshahrani N.Z., Alzahrani F., Alarifi A.M., Algethami M.R., Alhumam M.N., Ayied H.A.M., Awan A.Z., Almutairi A.F., Bamakhrama S.A., Almushari B.S., Sah R. (2022). Assessment of knowledge of monkeypox viral infection among the general population in Saudi Arabia. Pathogens.

[8] Alshahrani N.Z. (2022). Knowledge and attitude regarding monkeypox virus among physicians in Saudi Arabia, a cross-sectional study.

[9] Al-Tawfiq J.A., Barry M., Memish Z.A. (2022). International outbreaks of Monkeypox virus infection with no established travel: a public health concern with significant knowledge gap. Trav. Med. Infect. Dis..

[10] Visher E. (2021). The three Ts of virulence evolution during zoonotic emergence. Proc. Biol. Sci..

[11] Ministry Of Health Saudi Arabia (2022). Ministry of health Saudi Arabia. https://www.moh.gov.sa/en/Pages/Default.aspx.

[12] (Jul. 25, 2022). Treatment information for healthcare professionals | monkeypox | poxvirus | CDC. https://www.cdc.gov/poxvirus/monkeypox/clinicians/treatment.html.

